# The endocast of the Night Parrot (*Pezoporus occidentalis*) reveals insights into its sensory ecology and the evolution of nocturnality in birds

**DOI:** 10.1038/s41598-020-65156-0

**Published:** 2020-06-09

**Authors:** Andrew N. Iwaniuk, Aubrey R. Keirnan, Heather Janetzki, Karine Mardon, Stephen Murphy, Nicholas P. Leseberg, Vera Weisbecker

**Affiliations:** 1 Department of Neuroscience, University of Lethbridge, Lethbridge, AB Canada; 2 School of Biological Sciences, University of Queensland, St. Lucia, QLD Australia; 3 Queensland Museum, South Brisbane, QLD Australia; 4 Centre for Advanced Imaging, University of Queensland, St. Lucia, QLD Australia; 5 School of Earth and Environmental Sciences, University of Queensland, St. Lucia, QLD Australia; 6 College of Science and Engineering, Flinders University, GPO 2100 Adelaide, SA Australia

**Keywords:** Zoology, Evolution, Neuroscience

## Abstract

The Night Parrot (*Pezoporus occidentalis*) is a rare, nocturnal parrot species that has largely escaped scientific investigation due to its behaviour and habitat preferences. Recent field studies have revealed some insights into Night Parrot behaviour, but nothing is known of its sensory abilities. Here, we used μCT scans of an intact Night Parrot specimen to determine if its visual system shares similarities with other nocturnal species. The endocast of the Night Parrot revealed relatively small optic lobes and optic foramina, especially compared with closely related grass parakeets, but no apparent differences in orbit dimensions. Our data suggests that the Night Parrot likely has lower visual acuity than most other parrots, including its congener, the Eastern Ground Parrot (*P. wallicus*). We propose that the visual system of the Night Parrot might represent a compromise between the need to see under low light conditions and the visual acuity required to detect predators, forage, and fly. Based on the endocast and optic foramen measurements, the Night Parrot fits into a common pattern of decreased retinal input to the optic lobes in birds that should be explored more thoroughly in extant and extinct species.

## Introduction

The Night Parrot (*Pezoporus occidentalis*) is considered to be one of the world’s most elusive birds^1^. It is a small (100 g), highly cryptic, nocturnal parrot that lives only in the arid interior of Australia. Only 25 scientific specimens were collected between 1845 and around 1875, after which confirmed reports of living birds were absent for more than a century, despite an enormous potential range covering the majority of interior Australia^2^ and intense search efforts by several expeditions^1^. In 1990 and 2006, individual Night Parrot bodies were discovered^3,4^, but it was not until 2013 that photos of live birds and the discovery of a population in southwest Queensland enabled the first scientific study of this species^5^. Prior to 2013, all that was known about Night Parrot behaviour and ecology was based on anecdotal reports or inference^1^. The recent efforts of several researchers have since yielded data on Night Parrot movements^6^, vocalizations^7^, breeding behaviour^8^, a better understanding of habitat and dietary requirements^2,6^ and new populations discovered elsewhere in Australia^1,9^. Understanding more about the behaviour and ecology of this unique species is critical for species management throughout their range^1,2^, but the highly elusive behaviour of Night Parrots presents a major challenge to filling in knowledge gaps based on fieldwork alone.

One of the characteristic features of the Night Parrot is nocturnality. Adopting a nocturnal lifestyle is generally associated with substantial changes in eye and brain morphology in birds^10–12^. In some species, like owls and nightjars, the eye becomes enlarged, retinal anatomy changes in order to capture as many photons as possible, and visual processing areas in the brain are expanded^11,12^. In other species, however, the dependence on vision decreases in favour of using other senses, such as hearing, touch and/or smell, and the eyes and visual regions of the brain become smaller^13–15^. In the only other nocturnal parrot, the Kakapo (*Strigops habroptilus*), eye size and shape do not differ from diurnal parrots, but the optic tectum, the primary target of retinal ganglion cells, is greatly reduced in size^16^. The shrinkage of the Kakapo optic tectum coincides with a decrease in size of the optic foramen, which houses the optic nerve, as well as fewer retinal ganglion cells^16^. These anatomical changes result in the Kakapo possessing a visual system with a greater ability to capture photons under low light (higher sensitivity), but a relatively poor ability to discriminate among visual stimuli (lower acuity) compared to diurnal parrots^17^. Assessing whether the Night Parrot shares a similar visual system to the Kakapo would yield new insights into Night Parrot behaviour, including a better understanding of how they perceive their habitat.

Detailed study of the Night Parrot eye and brain anatomy is not possible due to the extremely limited number of living individuals and lack of fluid preserved museum specimens. However, much information regarding the Night Parrot’s visual system can be gleaned from the skull. For example, the optic foramen is well defined in parrots^18^ and reflects optic nerve size^16,18^ and orbital measurements can approximate eye size^19^. Digital endocasts (three dimensional reconstructions of the brain based on μCT scanning of skulls) have also been useful in assessing the sensory ecology of extinct birds^20–23^. In particular, the surface area of the optic lobes reflects the volume of the underlying optic tectum^24^, the midbrain region that receives the majority of retinal projections in birds^25^. The optic tectum is also the region that undergoes the greatest reduction in size in other nocturnal birds^15,21,26^, including the Kakapo^16^. Thus, the relative size of the optic foramen, orbits, and optic lobes of the Night Parrot could provide insights into its visual abilities.

**Figure 1:**
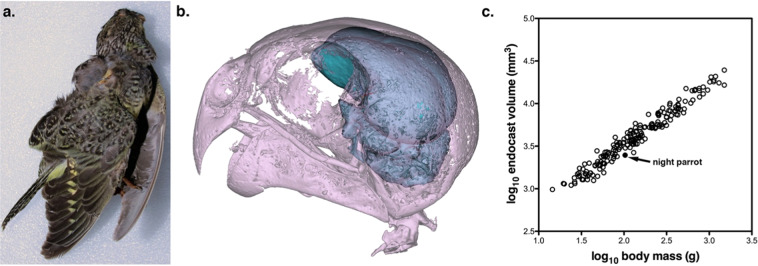
(**a**) A *photo* of the Night Parrot (*Pezoporus occidentalis*) specimen scanned in this study (QM O.29055). (**b)** The digitally reconstructed skull and endocast of the same specimen is shown. (**c)** A log-transformed scatterplot of endocranial volume plotted against body mass for 180 parrot species as well as the value obtained for the Night Parrot based on the reconstructed endocast.

Here, we used μCT scans of the only Night Parrot skull known to be intact^3^ (Fig. 1a) to quantify the dimensions of the orbits, optic foramen and optic lobes in comparison with other parrot species. Specifically, we predicted that the Night Parrot would have reduced optic lobes and smaller optic foramina, similar to the Kakapo^16^. However, we expected a reduction of lesser magnitude than what was found in the Kakapo because Night Parrots fly considerable distances between roosting and feeding areas^6^, probably making them more dependent on vision^27^.

## Results

**Figure 2:**
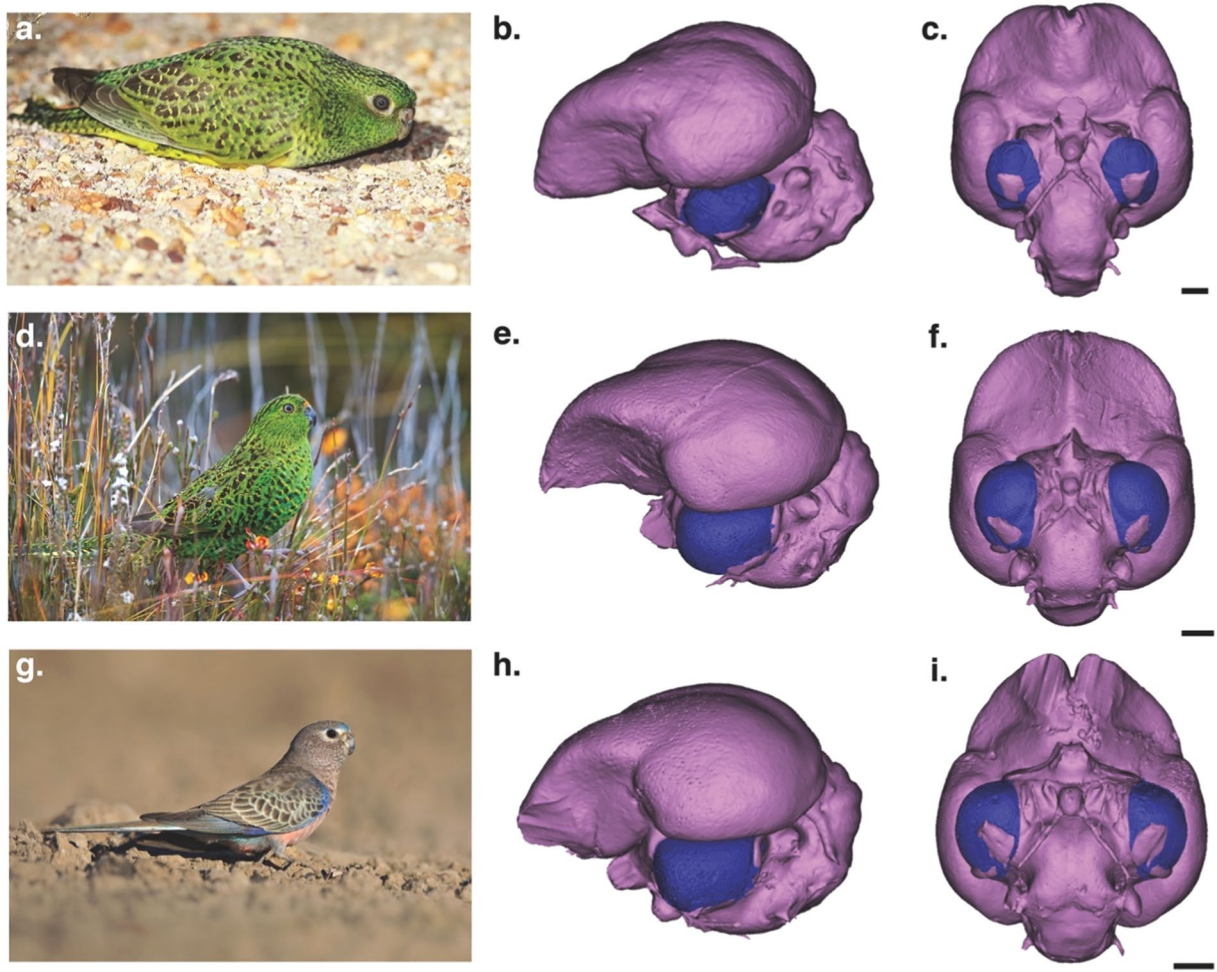
(**a**) A rare photo of a live Night Parrot (*Pezoporus occidentalis*) taken by S. Murphy; (**b)** Lateral view of the Night Parrot endocast with the optic lobe shown in blue; (**c)** Ventral view of the Night Parrot endocast; (**d)** A photo of the diurnal Eastern Ground Parrot (*Pezoporus wallicus*) provided by L. Ross; (**e)** Lateral *view* of the Ground Parrot endocast with the optic lobe shown in blue; (**f)** Ventral view of the Ground Parrot endocast; (**g)** a photo of the crespuscularly active Bourke’s Parrot (*Neopsephotus bourkii*) provided by D. Paton; (**h)** lateral view of the Bourke’s Parrot endocast with the optic lobe shown in blue; and (**i)** ventral view of the Bourke’s Parrot endocast. On each endocast, the optic lobes are shown in blue. All scale bars = 2 mm.

The μCT scans of the Night Parrot specimen (Fig. 1a) revealed some fragmentary material on the outside of the skull, but the braincase and orbits were entirely intact, allowing us to complete measurements of both the orbits and the endocast (Fig. 1b). Overall, the endocast had a volume of 2,478.07 mm^2^, which relative to body mass is typical of other small parrots (Fig. 1c) and similar to that of the Eastern Ground Parrot (*Pezoporus wallicus*). In terms of morphology, however, the optic lobes of the Night Parrot (Fig. 2a) appeared to be unusually small (Fig. 2b,c), especially compared with its congener and closest relative within the sample, the Eastern Ground Parrot (Fig. 2d–f), and the Bourke’s Parrot (*Neopsephotus bourkii*, Fig. 2g–i), a closely related grass parakeet^28^ that lives in similar habitat to the Night Parrot and is often active at dusk^29,30^.

**Figure 3:**
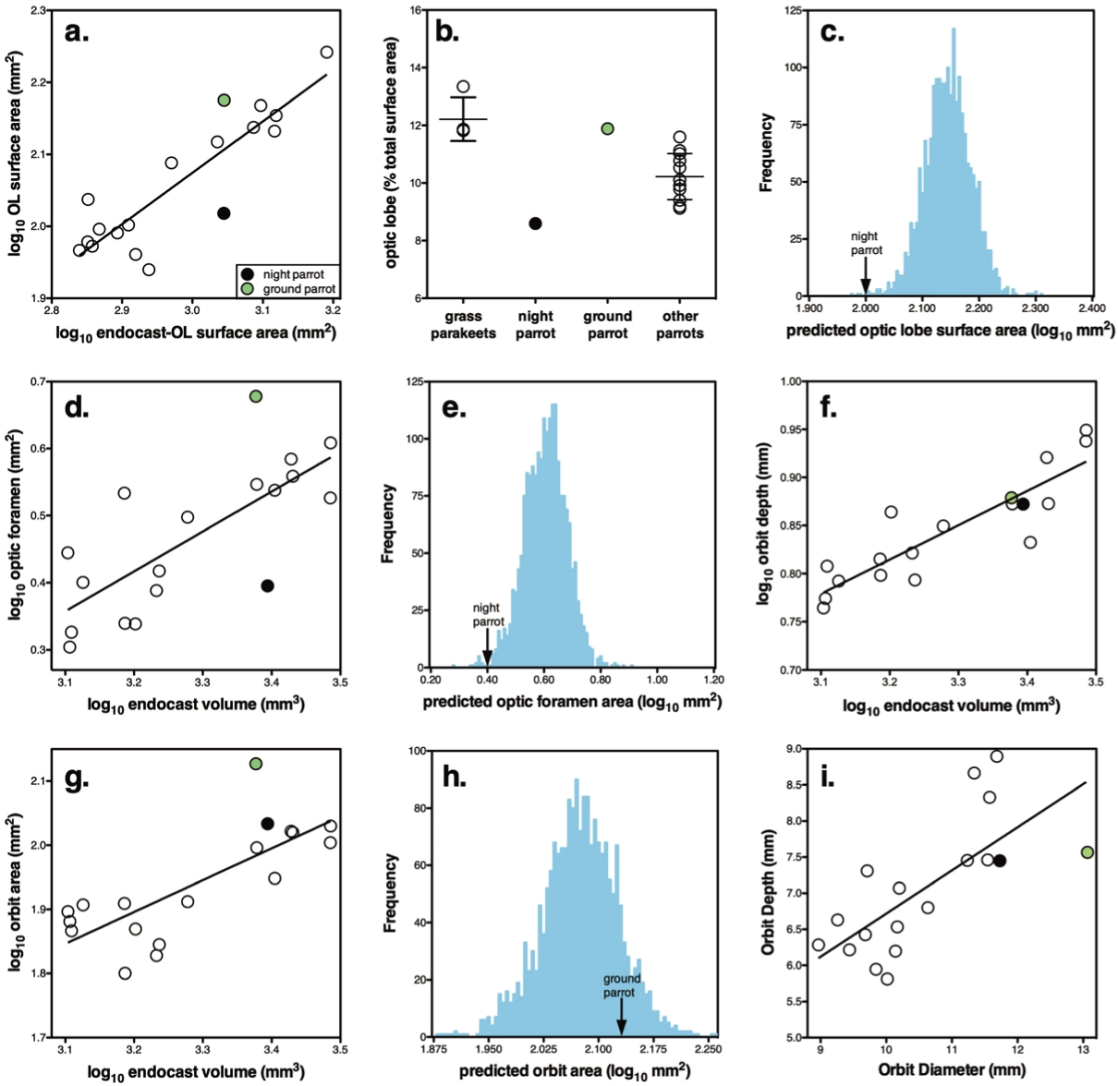
Scatterplots of the quantitative measurements of the skulls and endocasts of the 18 parrot species examined, and accompanying posterior probability distributions. In all scatterplots, the Night Parrot (*Pezoporus occidentalis*) is shown in black, the Ground Parrot (*Pezoporus wallicus*) in green and all other species in white. The solid lines indicate the least-squares linear regression lines. The arrows in the posterior probability distributions indicate the observed values of individual species. The plots are as follows: (**a)** log-transformed optic lobe surface area plotted against endocast surface area minus optic lobe surface area; (**b)** optic lobe surface area expressed as a percentage of endocast surface area; (**c)** the posterior probability distribution of the predicted surface area of the optic lobes of the Night Parrot based on phylogeny and allometric relationship with endocast-optic lobe surface area; (**d)** log-transformed optic foramen area plotted against endocast volume; (**e)** the posterior probability distribution of the predicted optic foramen area of the Night Parrot based on phylogeny and allometric relationship with endocast volume; (**f)** log-transformed orbit depth plotted against endocast volume; (**g)** log-transformed orbit area plotted against endocast volume; (**h)** the posterior probability distribution of the predicted orbit area of the Ground Parrot based on phylogeny and allometric relationship with endocast volume; and (**i**) orbit depth plotted against orbit diameter. Note that in (**b)**, the grass parakeets (*Neophema*, *Neopsephotus*) are shown separately to help illustrate the difference between the Night Parrot and other members of the tribe Pezoporini.

Compared with total endocast surface area, the Night Parrot had the smallest optic lobes of any of the species we examined (Fig. 3a,b). Although the Purple-crowned Lorikeet (*Glosspsitta porphyrocephala*) also has small optic lobes, when expressed as a percentage of total endocast surface area, the Night Parrot had the lowest value (Fig. 3b). More importantly, the relative size of the Night Parrot’s optic lobes contrasts greatly with that of its relatives, the grass parakeets (*Neophema*, *Neopsephotus*), and its congener the Eastern Ground Parrot, which had the largest optic lobes relative to endocast surface area (Fig. 3a,b). As shown in Fig. 3c, the optic lobes of the Night Parrot falls outside of the 95% interval of our phylogenetically informed posterior probability distributions. In fact, it exceeds the 99% interval, indicating that the surface area of the Night Parrot’s optic lobes is far below that predicted by its total endocast surface area.

The Night Parrot also had the smallest optic foramen relative to endocast volume (Fig. 3d). In fact, the optic foramen of the Night Parrot was similar in absolute size to that of the Budgerigar (*Melopsittacus undulatus*), a species with an endocast volume 2/3 that of the Night Parrot. At the other end of the spectrum, the Eastern Ground Parrot had an optic foramen that was almost 2x that of the Night Parrot even though they share similar endocast volumes (Fig. 3d). The relatively small size of the Night Parrot’s optic foramen is also supported by the posterior probability distribution (Fig. 3e).

Unlike the optic lobes and optic foramen, the Night Parrot did not differ in relative orbit area or depth from other parrots (Fig. 3f–i). Although the Ground Parrot did appear to have enlarged orbit area, relative to endocast volume (Fig. 3g), it did not fall outside of the 95% credible interval (Fig. 3h).

## Discussion

Overall, the Night Parrot has undergone a decrease in relative optic lobe size, at least compared with the closely related grass parakeets, and a decrease in relative optic foramen diameter compared with all other parrots examined. This indicates that the Night Parrot has evolved significant changes in its visual system anatomy, likely in response to its nocturnal activity. Although our conclusions are based on a single Night Parrot specimen, this is one of the rarest species in ornithological collections worldwide^1,31^. No osteological specimens appear to exist^1,3,31^ and the preparation of study skins involves damage to or removal of the skull^32^. The ‘mummified’ specimen^3^ is therefore the only Night Parrot specimen known to have an intact skull. Further, for species in which we measured three specimens, intraspecific coefficients of variation were 0.06-0.11 across the optic lobe and optic foramen measurements, suggesting that intraspecific variation is relatively low. If the Night Parrot’s optic lobe area and optic foramen diameter are underestimated by 11%, the observed values would still fall outside of the 95% credibility intervals in Fig. 3c,e. Thus, based upon the available data, we are reasonably confident that the Night Parrot has undergone reductions in the optic lobes and optic foramen, especially in comparison to the Eastern Ground Parrot and other grass parakeets.

Despite the reductions in optic lobes and foramina, the Night Parrot did not differ in orbit size from the other species examined. Eye shape, corneal diameter and retinal morphology are often better predictors of low light vision than eye size^10,18,19^. For example, the nocturnal Kakapo does not differ from other parrots in eye size or shape, but does have a higher density of photoreceptors and fewer retinal ganglion cells^16^. Currently, it is not possible to estimate photoreceptor density in the Night Parrot because a fluid preserved specimen does not exist and capturing one to preserve appropriately is not a viable option. However, the optic nerve is comprised primarily of retinal ganglion cell axons and the size of the optic foramen closely approximates that of the optic nerve in most birds, including parrots^16,18^. The relatively small optic foramina of the Night Parrot therefore reflects smaller optic nerves and, by extension, fewer retinal ganglion cells in the retina. If this assumption is correct, the Night Parrot likely has lower visual acuity than the other parrot species examined because the spatial resolving power of the eye is a product of retinal ganglion cell density and eye size^33^.

Corroborating support for fewer retinal ganglion cells in the Night Parrot is provided by the reduced optic lobes. The optic tectum receives the majority of the retinal efferents in most birds^25^ and species with fewer retinal ganglion cells also have relatively small optic tectum volumes^16,26,34^. Although the optic lobes house more than just the optic tectum, there is a strong correlation between optic tectum volume and the surface of the optic lobes^24^. Thus, the relatively small optic lobes of the Night Parrot likely reflect a decrease in optic tectum size and go hand in hand with smaller optic foramina.

A potential consequence of smaller optic foramina and optic lobes and fewer retinal ganglion cells is a decrease in visual acuity. Lower visual acuity is typically a consequence of living in scotopic (i.e., low light) environments^35^. In order to see effectively under scotopic conditions, the eye needs to enhance its sensitivity and this comes at the expense of visual acuity^35^. For example, in owls, and to a lesser extent Kakapo, a high population of photoreceptors converge on a lower number of retinal ganglion cells to improve sensitivity at the expense of acuity^16,36,37^. Based on our data, we suggest that the visual acuity of the Night Parrot is lower than that of other parrots, especially the closely related Eastern Ground Parrot and other grass parakeets. Having less acute vision is unlikely to be a hindrance to Night Parrots because they prefer open habitats with few, if any trees^1,6^, and the risk of flying into natural obstacles is low. Lower visual acuity and flying at night could, however, increase the risk of mortality arising from anthropogenic obstacles, such as fences, that are immobile and low contrast^38^. This is likely one of the primary reasons that owls and other nocturnal birds frequently become entangled on barbed-wire fences^39–41^. A survey of bird casualties due to barbed-wire fencing in Diamantina National Park, where Night Parrots also occur^42^, revealed that at least half of the species entangled are nocturnal or active at dusk^43^. More importantly, a decapitated Night Parrot was also found below a stretch of barbed-wire fencing on the park^4^ and the species most commonly killed as a result of fence strike was the crepuscularly active Bourke’s Parrot^43^. If we correct in our conclusion that the Night Parrot has lower visual acuity than diurnal parrots, barbed-wire fences could pose a significant hazard to the Night Parrot throughout their range^1^.

Although our interpretation of the Night Parrot endocast is somewhat speculative, the reduction of the optic foramina and lobes corroborates a more generalized relationship between brain morphology and nocturnality in birds. Overall, nocturnal birds have significantly smaller optic foramina than diurnal birds^18^. Many nocturnal birds also have relatively small optic tectum volumes ^15,16,26,44^. Similar reductions in the optic lobes and foramina are also reported in extinct birds, such as the elephantbirds (*Aepyornis* spp.)^21^ and Hawaiian ‘mole-duck’ (*Talpanas lippa*)^45^, both of which were interpreted as evidence of nocturnal behaviour. The Night Parrot adds to this generalized pattern of reduced optic foramina/nerves and optic lobes/tectum in nocturnal birds. We therefore emphasize that quantitative analyses of endocasts and cranial nerves may have significant potential in determining the activity pattern and sensory abilities of extinct and critically endangered bird species in ways that have thus far been largely overlooked.

## Materials and Methods

### Ethics Statement

No animals were collected for use in this study. All measurements were made from specimens housed at the Queensland Museum (see Table 1).

**Table 1:** The data collected for all 18 species examined in this study, including sample sizes (n) and specimen numbers. The data columns are as follows: ECV – endocranial volume (mm^3^), Brain SA – brain surface area (mm^2^), OLSA – optic lobe surface area (mm^2^), OF – optic foramen area (mm^2^), Orbit A – orbit area (mm^2^), and Orbit D – orbit depth (mm).

Species		n	Specimen number	ECV	Brain SA	OLSA	OF	Orbit A	Orbit D
*Cyanoramphus auriceps*	Yellow-crowned Parakeet	1	QMO.28238	2537.98	1726.97	174.54	3.45	108.05	10.63
*Glossopsitta concinna*	Musk Lorikeet	1	QMO.28231	3057.04	1443.83	135.66	3.36	78.82	11.34
*Glossopsitta* *porphyrocephala*	Purple-crowned Lorikeet	1	QMO.28574	1724.80	954.34	87.06	2.62	88.78	9.44
*Glossopsitta* *pusilla*	Little Lorikeet	1	QMO.12719	1537.40	880.31	97.94	2.19	80.76	8.97
*Melopsittacus undulatus*	Budgerigar	1	QMO.31840	1708.90	1053.00	96.75	2.45	81.18	9.26
*Neopsephotus bourkii*	Bourke’s Parrot	2	QMO.28232, QMO.28399	1278.11	806.35	95.49	2.02	67.30	9.84
*Neophema elegans*	Elegant Parrot	3	QMO.28276, QMO.28277, QMO.28291	1335.60	838.36	99.32	2.52	63.12	10.14
*Neophema pulchella*	Turquoise Parrot	3	QMO.28290, QMO.28589, QMO.28296	1269.61	822.30	109.03	2.79	133.99	10.02
*Neophema splendida*	Scarlet-chested Parrot	1	QMO.28293	1285.16	783.46	92.61	2.12	81.68	9.68
*Pezoporus occidentalis*	Night Parrot	1	QMO.29055	2478.07	1212.91	104.25	2.49	42.96	11.73
*Pezoporus wallicus*	Eastern Ground Parrot	1	QMO.28716	2382.71	1259.38	149.70	4.77	76.08	13.06
*Platycercus adscitus*	Pale-headed Rosella	1	QMO.31746	2680.79	1358.34	137.29	3.84	100.95	11.58
*Platycercus eximius*	Eastern Rosella	1	QMO.12720	2696.65	1397.49	147.20	3.62	107.17	11.55
*Platycercus icterotis*	Western Rosella	1	QMO.28307	2389.30	1216.22	131.02	3.52	104.66	11.24
*Psephotus haematonotus*	Red-rumped Parrot	1	QMO.28294	1897.10	1056.35	122.53	3.15	99.10	10.20
*Psephotus* *varius*	Mulga Parrot	1	QMO.16667	1534.53	911.62	100.46	3.42	105.18	10.17
*Psitteuteles versicolor*	Varied Lorikeet	1	QMO.12024	1592.65	921.98	91.41	2.18	74.02	9.71
*Trichoglossus chlorolepidotus*	Scaly-breasted Lorikeet	1	QMO.32344	3059.63	1457.50	142.60	4.06	108.05	11.68

### Specimens

To examine the anatomy of the endocast and orbits of the Night Parrot, we μCT scanned the specimen that was found in southwest Queensland in 1990^3^ (Fig. 1a), the only specimen known to have an intact skull. For comparison, we scanned a total of 22 specimens representing 17 other parrot species. Within the monophyletic clade Pezoporini^46^, to which the Night Parrot belongs^28^, we examined several grass parakeets (*Neophema* spp.), Bourke’s Parrot and the congeneric Eastern Ground Parrot. Bourke’s Parrots are frequently active after sunset^29,30^ and differ in vision and photoreceptor densities from exclusively diurnal parrot species^47,48^. The Ground Parrot shares similar terrestrial habits to the Night Parrot, but is diurnal and prefers coastal heathlands and sedgelands rather than arid interior regions^30,49,50^. The other 15 species examined were selected based on availability, size and phylogenetic distribution so that we had a sufficient range of sizes to calculate allometric relationships and had species representing clades other than Pezoporini within the Psittaciformes^46^.

**Figure 4:**
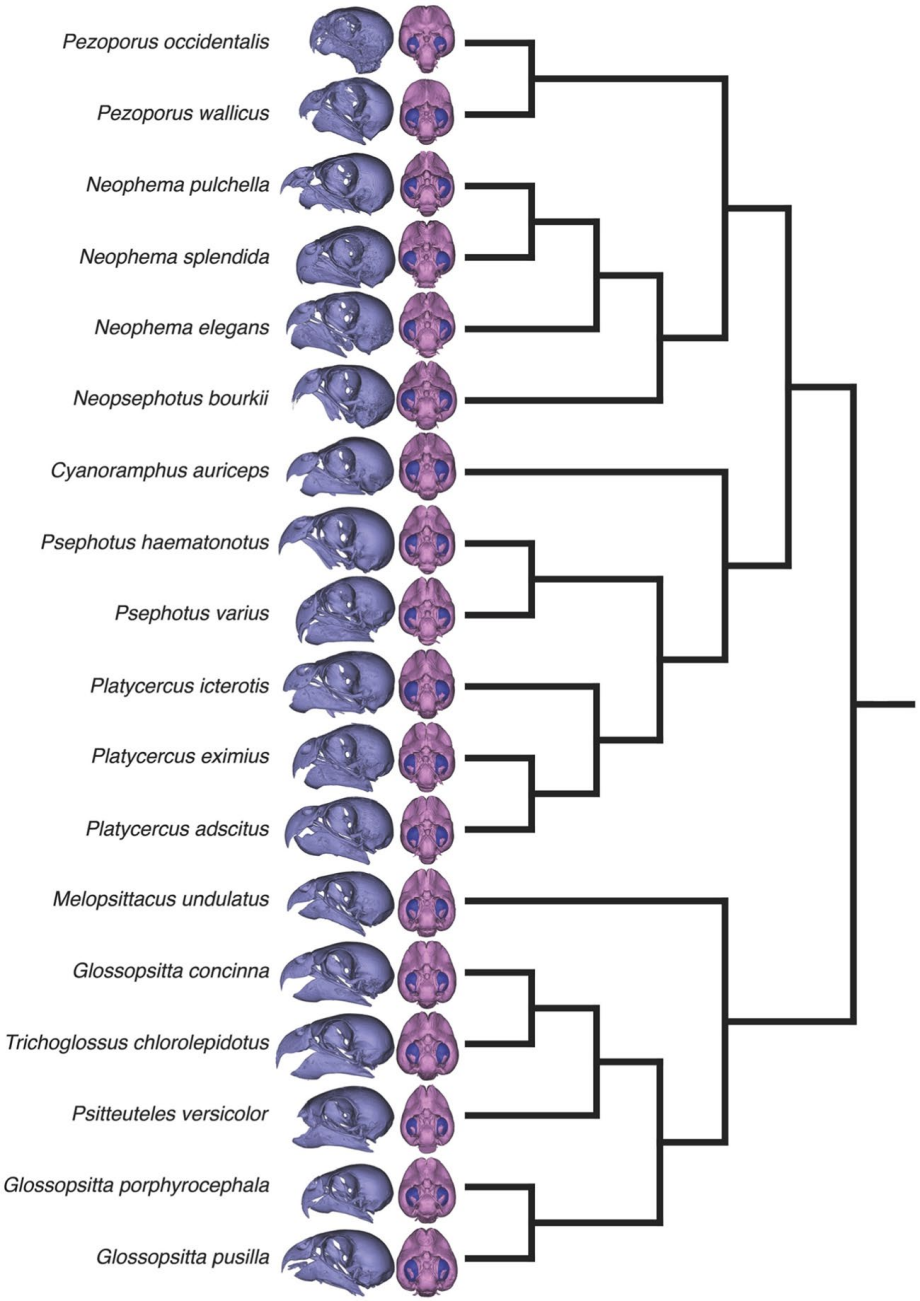
A phylogeny of the 18 parrot species examined in this study. The phylogeny was compiled from recent studies^28,53^. For each species, a digital reconstruction of the skull and a ventral view of the endocast is provided, with the optic lobes shown in blue. Note that the skulls and endocasts are not to scale.

The skulls were scanned using high-resolution X-ray computed tomographic scans (μCT) from a Siemens Inveon PET/CT scanner at the Centre for Advanced Imaging, University of Queensland. The resulting DICOM files were imported into Mimics (v18.0, Materialise NV) and skull ‘masks’ were produced through thresholding (Fig. 4). Segmenting the endocast was done slice-by-slice ensuring that foramina and fenestrae were segmented consistently across specimens^51^. A complete list of the species, specimen numbers and data are provided in Table 1. All original DICOM scans with associated acquisition parameters, as well as the surface files of cranial reconstructions and endocasts, can be found on MorphoSource (Project P453).

### Measurements

3D reconstructions of the skulls were used for measuring the surface area of the orbit, optic foramen and foramen magnum as well as the orbit depth within Mimics. All of the quantitative measurements are provided in Table 1. Orbit diameter was assessed through use of the ellipse tool. The ellipse was placed to touch the left, right, upper, and lower extremes of the orbital rim, and the surface area of the resulting (mostly circular) ellipse was determined. Similarly, the ellipse tool was used to outline the optic foramen and determine its area. Orbit depth was measured from the dorsal rim of the optic foramen to the center of the plane described by the ellipse fitted across the orbit, the same ellipse that was used to measure orbit circumference (see Figure S1 in supplementary information).

Acquiring the surface area of the optic lobes required a stepwise approach. The trigeminal nerve and blood vessel located on the surface of the optic lobe were digitally removed to avoid overestimates of optic lobe area. After this, the optic lobes were digitally dissected from the rest of the endocast and data was recorded for each side and then averaged. As the total surface area of the lobes included two sides where they had been cut from the rest of the endocast, the ellipse tool was used to measure these areas, so they could be subtracted from this total. The original left and right measurements are provided in the supplementary information (Table S1). For species represented by more than one specimen, data were averaged for analysis (Table 1).

Mesh volumes, using the “optimal resolution” setting in Mimics, were used to facilitate the dissection of the optic lobes (Figure S2 in supplementary information). The conversion of mesh volumes from voxel volumes produces slight discrepancies between the voxels and the mesh outlines, potentially leading to very small deviations of voxel-based vs. mesh-based volume estimates (Figure S2). Relative to the volumes measured, this effect is very small, identical across dissections, and does not result in distortion of the overall shape. It was therefore deemed negligible as a source of error.

### Statistical analysis

We first plotted endocast volume against body mass of the Night Parrot and a large dataset of endocranial volumes of 180 other parrot species^52^. Body mass for the Night Parrot is the average of two specimens that were captured for a GPS tracking study^6^ and for other species from^52^. We then used a phylogeny informed statistical approach to test whether the Night Parrot deviated from allometric relationships for optic lobe surface area, optic foramen area and orbit measurements. The phylogeny was constructed from published papers^28,53^ using Mesquite^54^ (Fig. 4). Because the phylogeny was constructed from different sources, we set all branch lengths to one. To then evaluate whether the Night Parrot differed in the relative size of the optic lobes, optic foramen, and orbits, we generated posterior probability distributions of the expected values using a Bayesian Markov Chain Monte Carlo approach across the phylogeny, following the procedures outlined in Nunn and Zhu^55^ and performed in R^56^. We ran 200,100 iterations with a burnin rate of 100 and a thin rate of 100 with endocast volume as the predictor variable to generate posterior probability distributions of 2,000 values of the predicted sizes of the optic lobes, optic foramen, and orbit dimensions of the Night Parrot.

## Supplementary information

Supplementary Information.

Supplementary Information.
