# Author Correction: The endocast of the Night Parrot (*Pezoporus occidentalis*) reveals insights into its sensory ecology and the evolution of nocturnality in birds

**DOI:** 10.1038/s41598-020-70122-x

**Published:** 2020-07-28

**Authors:** Andrew N. Iwaniuk, Aubrey R. Keirnan, Heather Janetzki, Karine Mardon, Stephen Murphy, Nicholas P. Leseberg, Vera Weisbecker

**Affiliations:** 1 Department of Neuroscience, University of Lethbridge, Lethbridge, AB Canada; 2 School of Biological Sciences, University of Queensland, St. Lucia, QLD Australia; 3 Queensland Museum, South Brisbane, QLD Australia; 4 Centre for Advanced Imaging, University of Queensland, St. Lucia, QLD Australia; 5 School of Earth and Environmental Sciences, University of Queensland, St. Lucia, QLD Australia; 6 College of Science and Engineering, Flinders University, GPO 2100, Adelaide, SA Australia

Correction to: *Scientific Reports* https://doi.org/10.1038/s41598-020-65156-0, published online 09 June 2020

This Article contains errors in Table 1 where columns ‘Orbit A’ and ‘Orbit D’ should be named ‘Orbit Area’ and ‘Orbit Diameter’ respectively.

Furthermore, there are errors in column Orbit A where all data points given are incorrect.

Finally, a column entitled ‘Orbit Depth’ is omitted. The correct Table 1 is below.

**Table 1:** The data collected for all 18 species examined in this study, including sample sizes (n) and specimen numbers. The data columns are as follows.

Species		n	Specimen number	ECV	Brain SA	OLSA	OF	Orbit Area	Orbit Diameter	Orbit Depth
*Cyanoramphus auriceps*	Yellow-crowned Parakeet	1	QMO.28238	2537.98	1726.97	174.54	3.45	88.78	10.63	6.80
*Glossopsitta concinna*	Musk Lorikeet	1	QMO.28231	3057.04	1443.83	135.66	3.36	100.95	11.34	8.67
*Glossopsitta porphyrocephala*	Purple-crowned Lorikeet	1	QMO.28574	1724.80	954.34	87.06	2.62	69.98	9.44	6.22
*Glossopsitta pusilla*	Little Lorikeet	1	QMO.12719	1537.40	880.31	97.94	2.19	63.12	8.97	6.29
*Melopsittacus undulatus*	Budgerigar	1	QMO.31840	1708.90	1053.00	96.75	2.45	67.30	9.26	6.63
*Neopsephotus bourkii*	Bourke’s Parrot	2	QMO.28232, QMO.28399	1278.11	806.35	95.49	2.02	76.08	9.84	5.95
*Neophema elegans*	Elegant Parrot	3	QMO.28276, QMO.28277, QMO.28291	1335.60	838.36	99.32	2.52	80.76	10.14	6.20
*Neophema pulchella*	Turquoise Parrot	3	QMO.28290, QMO.28589, QMO.28296	1269.61	822.30	109.03	2.79	78.82	10.02	5.81
*Neophema splendida*	Scarlet-chested Parrot	1	QMO.28293	1285.16	783.46	92.61	2.12	73.56	9.68	6.43
*Pezoporus occidentalis*	Night Parrot	1	QMO.29055	2478.07	1212.91	104.25	2.49	108.05	11.73	7.45
*Pezoporus wallicus*	Eastern Ground Parrot	1	QMO.28716	2382.71	1259.38	149.70	4.77	133.99	13.06	7.57
*Platycercus adscitus*	Pale-headed Rosella	1	QMO.31746	2680.79	1358.34	137.29	3.84	105.18	11.58	8.33
*Platycercus eximius*	Eastern Rosella	1	QMO.12720	2696.65	1397.49	147.20	3.62	104.66	11.55	7.46
*Platycercus icterotis*	Western Rosella	1	QMO.28307	2389.30	1216.22	131.02	3.52	99.10	11.24	7.46
*Psephotus haematonotus*	Red-rumped Parrot	1	QMO.28294	1897.10	1056.35	122.53	3.15	81.68	10.20	7.07
*Psephotus varius*	Mulga Parrot	1	QMO.16667	1534.53	911.62	100.46	3.42	81.17	10.17	6.54
*Psitteuteles versicolor*	Varied Lorikeet	1	QMO.12024	1592.65	921.98	91.41	2.18	74.02	9.71	7.31
*Trichoglossus chlorolepidotus*	Scaly-breasted Lorikeet	1	QMO.32344	3059.63	1457.50	142.60	4.06	107.17	11.68	8.90

The data columns are as follows: ECV – endocranial volume (mm^3^), Brain SA – brain surface area (mm^2^), OLSA – optic lobe surface area (mm^2^), OF – optic foramen area (mm^2^), Orbit Area (mm^2^), Orbit Diameter (mm), and Orbit Depth (mm).

