# Supplementary material for: The endocast of the Night Parrot (Pezoporus occidentalis) reveals insights into its sensory ecology and the evolution of nocturnality in birds
Source: Sci Rep. 2020 Jun 9;10:9258. doi: 10.1038/s41598-020-65156-0 (PMC7283296; doi:10.1038/s41598-020-65156-0)
Supplement: Supplementary file 1 — Supplementary Information. [file 41598_2020_65156_MOESM1_ESM.pdf]

The endocast of the Night Parrot (*Pezoporus occidentalis*) reveals insights into its sensory ecology and the evolution of nocturnality in birds.

Andrew N. Iwaniuk[1], Aubrey R. Keirnan[2], Heather Janetzki[3], Karine Mardon[4], Stephen Murphy[5], Nicholas P. Leseberg[5], and Vera Weisbecker[2,6]

[1]Department of Neuroscience, University of Lethbridge, Lethbridge, AB, Canada

[2]School of Biological Sciences, University of Queensland, St. Lucia, QLD, Australia

[3]Queensland Museum, South Brisbane, QLD, Australia.

[4]Centre for Advanced Imaging, University of Queensland, St. Lucia, QLD, Australia

[5]School of Earth and Environmental Sciences, University of Queensland, St. Lucia, QLD, Australia

[6]College of Science and Engineering, Flinders University, GPO 2100, Adelaide, SA, Australia

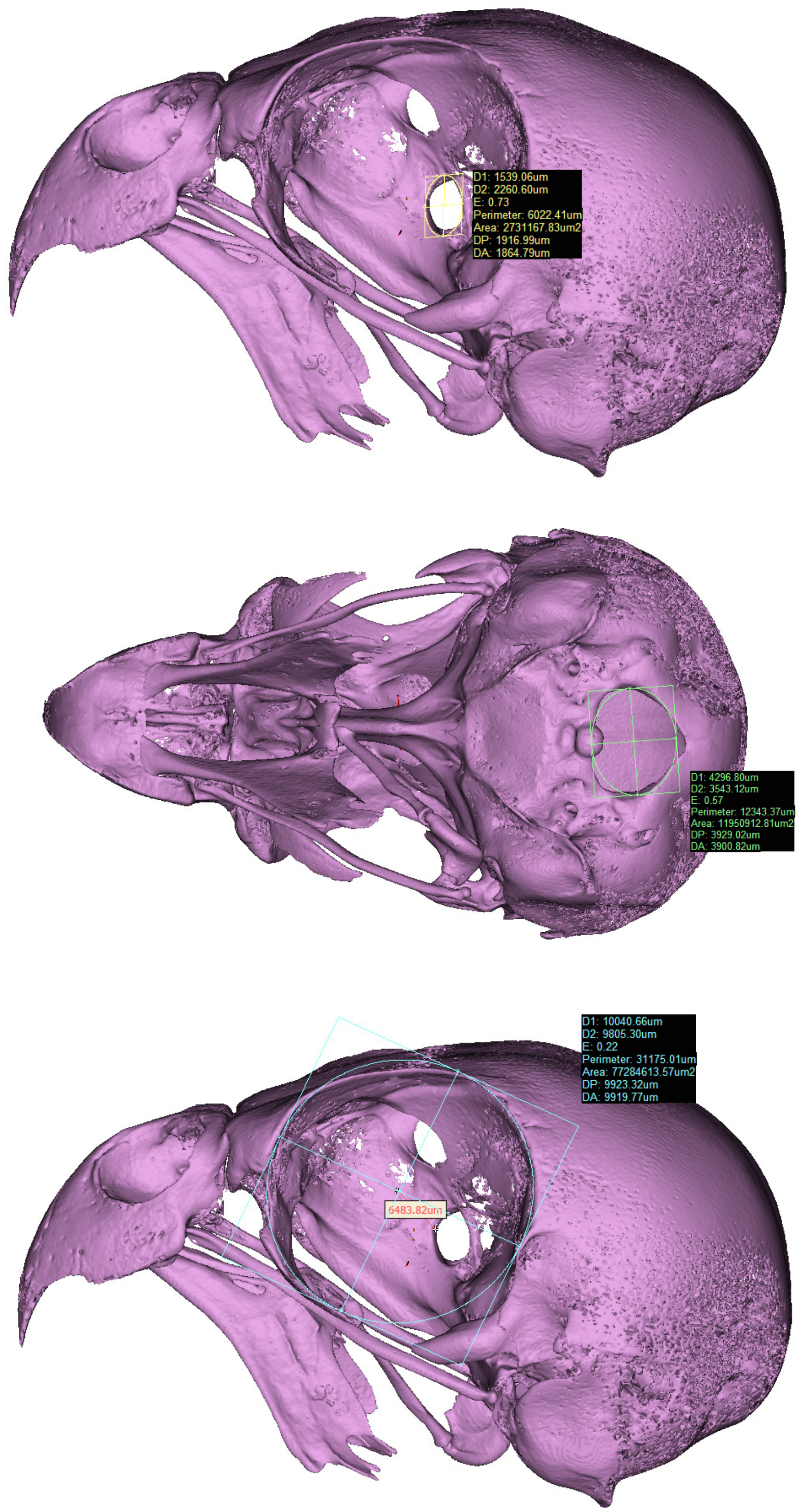

Supplementary Figure 1: Mimics software. Lateral views show the measurement technique for the surface area of the optic foramen (left) and the orbit (right) as well as the orbit depth while ventral view shows foramen magnum measurement.

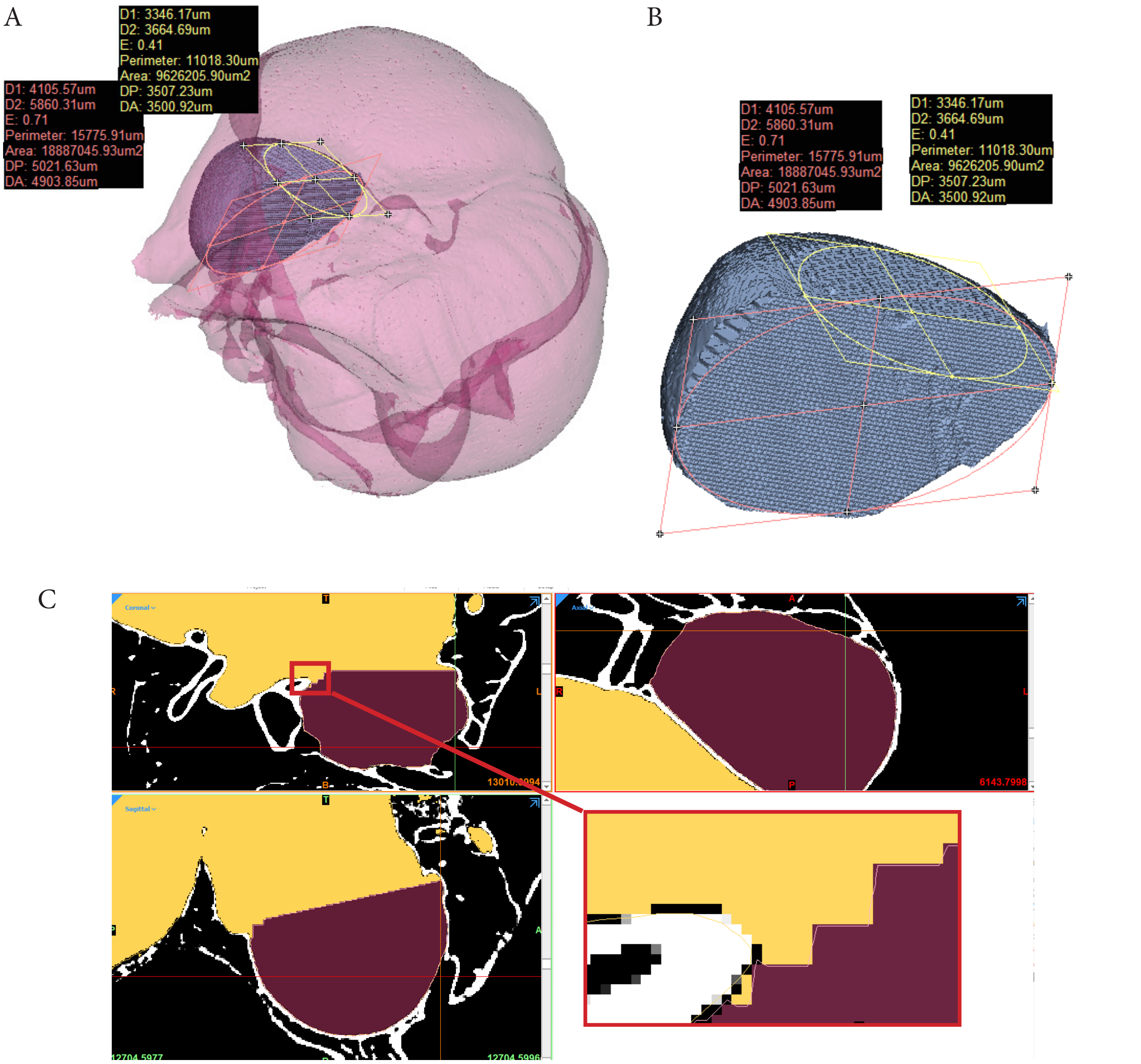

Supplementary Figure 2. A, Full endocranial cast of *Neophema elegans* on the right with transparency settings used to show the position of a digitally dissected optic lobe. B, a close-up image of the same lobe as well as the ellipses used to measure the regions which are not part of the optic lobe surface area. his included one full ellipse and one half ellipse, whose surfaces were then sub-tracted from the surface area of the dissected-out lobe area. C, Note that the dissection of volumes in 3D causes very minor, systematic (i.e. unbiased) discrepancies between the original voxel and derived mesh volumes. his is visible when comparing the pink contour lines of the 3D mesh of the right olfactory bulb with the dark red actual voxel representation, which can only seen at high magnifications (see inset on bottom right).
